# Evaluation of carbon tetrachloride fraction of *Actinodaphne angustifolia* Nees (Lauraceae) leaf extract for antioxidant, cytotoxic, thrombolytic and antidiarrheal properties

**DOI:** 10.1042/BSR20201110

**Published:** 2020-06-22

**Authors:** Mohammad Najim Uddin, Towsif Alam, Muhammad Azharul Islam, Tawhidul Amin Khan, Raihan Uz Zaman, Shofiul Azam, ATM Mostafa Kamal, Md. Jakaria

**Affiliations:** 1 Department of Pharmacy, Faculty of Science & Engineering, International Islamic University Chittagong, Chattogram 4318, Bangladesh; 2 Department of Thai Traditional Medicine, Faculty of Traditional Thai Medicine, Prince of Songkla University, Hat Yai, Songkhla 90112, Thailand; 3 Department of Applied Life Sciences and Integrated Bioscience, Graduate School, Konkuk University, Chungju 27478, Korea; 4 The Florey Institute of Neuroscience and Mental Health, The University of Melbourne, Parkville, VIC 3010, Australia

**Keywords:** Actinodaphne angustifolia Nees, Antidiarrheal activity, Antioxidant activity, Cytotoxic activity, Thrombolytic activity

## Abstract

*Actinodaphne angustifolia* Nees (Family: Lauraceae) is commonly used in folk medicine against urinary disorder and diabetes. The objective of the present study was to evaluate the antioxidant, cytotoxic, thrombolytic, and antidiarrheal activities of carbon tetrachloride (CCl4) fraction of leaves of *A. angustifolia* (CTFAA) in different experimental models. Antioxidant activity was evaluated by using qualitative and quantitative assays, while antidiarrheal effects assessed with castor oil-induced diarrheal models in mice. The clot lysis and brine shrimp lethality bioassay were used to investigate the thrombolytic and cytotoxic activities, respectively. CTFAA showed antioxidant effects in all qualitative and quantitative procedures. The fraction produced dose-dependent and significant (*P*<0.05 and *P*<0.01) activities in castor oil-induced diarrheal models. Moreover, CTFAA significantly (*P*<0.05) demonstrated a 15.29% clot lysis effect in the thrombolytic test, and the brine shrimp lethality assay LC_50_ value was 424.16 μg/ml bioassay. In conclusion, the current study showed CTFAA has significant antidiarrheal effects along with modest antioxidant and thrombolytic effects, and these data warrant further experiment to justify and include CTFAA as a supplement to mitigate the onset of diarrheal and cardiovascular disease.

## Introduction

Bangladesh is very rich in medicinal plants, and more than a thousand plants have been documented with prominent pharmacological efficiency [1]. Although advanced allopathic medicines are available, a vast majority (75–80%) of the population of this country still preferring traditional medicine as the first line of therapy for most of the diseases, because of affordability, availability, and safety [2]. It has been reported that natural remedies have a broad range of beneficial activities in the experimental studies [3–10]. Over the centuries, plant-based ailment has been used by different communities worldwide [11]. The local people of Bangladesh utilize medicinal plants as a primary source of their healthcare system to cure a large number of diseases [12]. The efficiency of plant-derived drugs mostly depends on their antioxidative property. In most disease cases, excessive reactive oxygen species generation plays as a major culprit; therefore, either neutralizing or controlling reactive oxygen species production is the best possibility to delay or cure several disease conditions. This hypothesis directs novel therapeutics researchers to target natural sources repeatedly.

Diarrhea is the frequent bowel movements with wet stool and abdominal cramps [13]. It is one of the most killer diseases in the world and contributing substantial pediatric morbidity and mortality [14], especially a threat for malnourished children in developing countries. Despite repeated effort has been given to eradicate this threat, yet the prevalence and incidence of diarrhea are high (approximately 7.1 million per year) [15]. Current therapy of diarrhea includes antibiotics, which sometimes aggravate adverse effects and no-antibiotic treatment also causes some common adverse effects like constipation [16]. Therefore, a safe and effective agent is being searched from plant origin that has always been an essential source of new drugs. The World Health Organization (WHO) is also encouraging the study and finding the treatment and prevention of diarrheal diseases using traditional medicines [17].

Thrombosis is a lethal disease, which relates to acute coronary disorders such as pulmonary emboli, deep vein thrombosis, strokes, heart failure, and venous thromboembolic disorders that account for sudden morbidity and mortality [18]. Thrombosis is also associated with the vascular blockade and while it is recovering, can cause a fatal consequence like cerebral or myocardial infarction and even leads to death [19]. They are currently using thrombolytic agents including tissue plasminogen activator (tPA), alteplase, anistreplase, urokinase, and streptokinase (SK) and recombinant tPA, although, highly effective treatment for thrombolysis. But these therapeutics are associated with severe adverse effects such as anaphylactic reaction, systemic fibrinolysis, hemorrhage, slow reperfusion rate, and frequent early occlusions [20]. An alternative option from traditional and herbal drugs is frequently being searched for their safety profile, thus, to overcome current adverse effects of this therapy based on the high importance of the thrombolytic agents. Several plants have already been documented as emerging and potential thrombolytic agents [21], which has encouraged to extend the search of potential thrombolytic agents from the plant source.

*Actinodaphne* is an Asian genus belongs to the family Lauraceae, bay laurel-related, which is a flowering plants class within the order Laurales [22]. This genus is important both pharmaceutically and medicinally. For instance, *A. hookeri* seed oil is used in rheumatic arthritis, and the bark is used in fracture, *A. lancifolia* has potential effect in the treatment of edema, arthritis, and overexertion [23–26]. *Actinodaphne angustifolia* Nees is a medium-sized evergreen tree and widely distributed in the forests of Chittagong, Chittagong Hill Tracts (CHT), Cox’s Bazar, Sylhet, and North Bengal regions of Bangladesh. Although a little information of pharmacological activity has documented, traditionally *A. angustifolia* has been used for treatments. Locally, the leaves are used in the treatment of urinary disorder and diabetes in Khagrachari district of CHT. A phytochemical investigation of the leaves has shown the presence of β-sitosterol, quercetin-3-O-rhamnoside, vitexin, friedelin, and hydrocarbons [27,28]. Presence of these phytochemicals suggests that *A. angustifolia* leaves might have potential antioxidant property and so the present study postulate that the carbon tetrachloride (CCl4) fraction of leaves of *A. angustifolia* (CTFAA) may have possible activity against thrombosis, diarrhea and oxidative stress.

## Materials and methods

### Plant and chemicals

Fresh leaves of *A. angustifolia* were collected from forest region of Rangamati, Bangladesh. The part of the plant was then identification by a taxonomist Prof. Shaikh Bokhtear Uddin, and a voucher specimen (Accession number: Kapran 034) was deposited to Chittagong herbarium for further reference. The leaves were air-dried, ground to powder and stored in a plastic vacuum container until further use. Methanol, *n*-hexane, carbon tetrachloride, dichloromethane, ethyl acetate, dimethyl sulfoxide (DMSO), Folin-Ciocalteu reagent, ascorbic acid, and castor oil were bought from Merck, India. 2,2-Diphenyl-1-picrylhydrazyl (DPPH) (Sigma–Aldrich), gallic acid (Ashland Chemical), and quercetin (Merck) were obtained from the local sources. Standard drugs SK, vincristine sulfate, and loperamide were bought from Beacon Pharmaceuticals, Bangladesh. All residual chemicals were of analytical grade.

### Extraction and fractionation

Approximately 2.710 kg of the powdered plant material was exhaustively macerated with 80% methanol and filtered. Then the filtrates were concentrated at vacuum rotary evaporator (Sterilin, U.K.) at 40°C for 24 h and a 110 g yield (4.06% w/w) of the crude extract obtained. The crude methanol extract was successively partitioned with *n*-hexane, carbon tetrachloride, dichloromethane, and ethyl acetate, and 0.68 g, 2.80 g, 2.35 g, and 64.4 mg of soluble fractions obtained, respectively [29]. The fractions were stored at 4°C in airtight containers until further analysis.

### Brine shrimp lethality bioassay

Brine shrimp lethality bioassay was performed to investigate the cytotoxicity of CTFAA [30]. Brine shrimp (*Artemia salina*) eggs were hatched in a conically shaped vessel (capacity = 1 l) containing artificial seawater (sea salt 38 g/l; adjusted pH 8.5). A divider was used to make two unequal compartments in the vessel. The eggs were sprinkled into the larger chamber, which was darkened, while the smaller compartment was illuminated. The vessel was placed at room temperature (25°C) under constant aeration. After 48 h, live nauplii free from eggshells were collected from the brighter portion of the hatching chamber. The fraction (25 mg) was dissolved in 5 ml of 1% aqueous DMSO solution to obtain a concentration of 5 mg/ml, which was subjected to serial dilution, getting different concentrations of 1000, 800, 500, 300, 200, and 100 µg/ml. Vincristine sulfate and 1% DMSO solution were used as positive and negative controls, respectively. Ten shrimp nauplii were transferred to each sample test tube. After 24 h incubation at 25°C, the number of viable nauplii was counted using a magnifying glass. The median lethal concentration (LC_50_) was then determined. The present study was an observational study where no surgery implicated, and the study was taken place at the Department of Pharmacy, International Islamic University Chittagong, Bangladesh. The study protocol was approved by the Planning and Development (P&D) Committee, Department of Pharmacy, International Islamic University Chittagong, Bangladesh (Pharm-P&D-87/56′33-18).

### Antioxidant activity

#### Determination of free radical scavenging activity

The stable DPPH was used for the estimation of free radical scavenging activity [31]. A 0.004% of DPPH (w/v) solution was prepared in methanol and 3 ml of that solution was added to different concentration of either extract or standard (500–15.625 µg/ml). The mixture was incubated at room temperature for 30 min, then absorbance taken at 517 nm by using a UV–visible spectrophotometer. The percentage of inhibition was calculated from [(*A*_0_ – *A*_1_)/*A*_0_] × 100, where *A*_0_ is the absorbance of the control and *A*_1_ is the absorbance of the test sample/standard.

#### Determination of reducing power capacity

The basis of reducing power capacity determination assay is the per cent of Fe (III) to Fe (II) transformation, where the endpoint is determined by the development of Perl’s Prussian blue color and UV-spectrum measured at 700 nm [32]. In brief, 1 ml of the test sample or standard (at concentrations of 500–15.625 µg/ml) was mixed with phosphate buffer (2.5 ml, 0.2 M, pH 6.6) and potassium ferricyanide (2.5 ml, 1% w/v). The mixture was incubated for 20 min at 50°C to complete the reaction. A portion (2.5 ml) of 10% (v/v) trichloroacetic acid (TCA) was added to the blend and centrifuged at 3000 rpm for 10 min at room temperature. The supernatant was collected and mixed with distilled water (2.5 ml) and FeCl_3_ (0.5 ml, 0.1% w/v), a visible Prussian blue color was seen, indicating the endpoint of the reaction. Absorbance was measured at 700 nm by using a UV–visible spectrophotometer against blank.

#### Determination of total phenolic content

The total phenolic content was determined by using the Folin-Ciocalteu reagent [33]. A 0.5 ml of the test sample (1 mg/ml) or standard (500–15.625 µg/ml) and 2.5 ml of Folin-Ciocalteu reagent (diluted ten times with water) was blended. Subsequently, 2 ml of Na_2_CO_3_ (7.5% w/v) was added at the time interval of 0.5 min until 8 min. The blend was incubated for 5 min at 50°C to complete the reaction and then cooled. Then the absorbance was evaluated at 760 nm by using a UV–visible spectrophotometer against blank. The experiment was conducted triplicate. Gallic acid standard curve was used to quantify total phenolic content, and the results were expressed as mg of gallic acid equivalent (GAE)/g of the dried fraction.

#### Determination of total flavonoid content

Total flavonoid content was determined by aluminum colorimetric assay [34]. One milliliter (1 ml) of the test sample (1 mg/ml) or standard (at different concentrations 100–12.5 µg/ml) was mixed with 3 ml of methanol, 0.2 ml of 10% AlCl_3_, 0.2 ml of 1 M potassium acetate and 5.6 ml of distilled water. The blend was incubated for 30 min at room temperature to complete the reaction. Then the absorbance was measured at 420 nm by using a UV–visible spectrophotometer against blank. The experiment was conducted triplicate. Quercetin standard curve was used to quantify total flavonoid contents, and the results were expressed as mg of quercetin equivalent (QE)/g of the dried fraction.

#### Determination of total flavonol content

Total flavonol content was determined by using quercetin as a reference compound [35]. One milliliter (1 ml) of test sample (1 mg/ml) or standard (at different concentrations 100–12.5 µg/ml) was mixed with 1 ml AlCl_3_ (20 mg/ml) and 3 ml sodium acetate (50 mg/ml). The blend was incubated for 2.5 h at room temperature to complete the reaction. Then the absorbance was measured at 440 nm by using a UV–visible spectrophotometer. The experiment was conducted in triplicates. Quercetin standard curve was used to quantify total flavonol contents, and the results were expressed as mg of quercetin equivalent (QE)/g of the dried fraction.

### Experimental animals

Swiss albino mice (both sexes) weighing approximately 30–35 g were used for this experiment. The mice were purchased from the animal research branch of the International Center for Diarrheal Disease and Research, Bangladesh (ICDDR, B) and were provided with a standard diet (ICDDR, B formulated) and clean water *ad libitum*. Acclimatization was done as per the previous published article [36]. Mice were kept in standard environmental conditions (55–60% relative humidity; 25 ± 2°C room temperature; 12 h light/dark cycle) for 1 week in the animal house for acclimation. The animals fasted overnight (13–14 h) before the experiments. All animal experiments were taken place at the Department Pharmacy, International Islamic University Chittagong (IIUC). Before conducting the animal works, an approval was taken from the developmental committee of the Department Pharmacy, International Islamic University Chittagong (IIUC). The study protocol was approved by the Planning and Development (P&D) Committee, Department of Pharmacy, International Islamic University Chittagong, Bangladesh (Pharm-P&D-87/56′33-18).

### Acute toxicity assessment

Mice were separated into four groups of five mice for each. They were fasted overnight and then orally administered with the CTFAA at the doses of 1000, 2000, and 3000 mg/kg, while the control group only received the vehicle. The mice were observed for any abnormal behavior such as sedation, respiratory distress, motor impairment, and hyperexcitability for 3 h. Furthermore, the incidence of mortality for each group was recorded up to 24 h after administration. Food and water were provided *ad libitum* [37].

### Thrombolytic activity

Tests for clot lysis were carried out as reported earlier [38]. 1.5 ml venous blood drawn from healthy human volunteers (Consent approval number: ECPD-IIUC-2018/04) was distributed in three different pre-weighed sterile microcentrifuge tubes (0.5 ml/tube) and incubated at 37°C for 45 min. After clot formation, serum was wholly removed without troubling the clot and each tube having clot was again weighed to define the clot weight (clot weight = weight of clot containing tube – the weight of tube alone). To each microcentrifuge tube containing pre-weighed clot, 100 μl of aqueous CTFAA, as a positive control, 100 μl of SK and as a negative non-thrombolytic control, 100 μl of distilled water were separately added. Then, the incubation of the tubes was done at 37°C for 90 min and noticed for clot lysis. After the completion of incubation, fluid released was removed, and tubes were again weighed to get the difference in weight after clot disruption. The difference obtained in weight taken before and after clot lysis was expressed as the percentage of clot lysis. The experiment was repeated ten times with the blood samples of 10 volunteers.

### Antidiarrheal activity

#### Castor oil-induced diarrhea

Castor oil-induced diarrhea was performed according to the previously described method [39]. The animals were divided into four groups of five mice for each. Three groups were treated with the CTFAA (150 and 300 mg/kg) and loperamide hydrochloride (5 mg/kg) orally. The control group was given 1% DMSO solution (10 ml/kg) orally. Later (30 min), each animal received 0.3 ml castor oil orally. After that, they were individually placed in cages where the floor was lined with white paper. The following parameters were observed for a period of 4 h, an onset of diarrhea, the total number of feces as well as the number of diarrheic feces excreted by the animals in 4 h, and the total weight of diarrheal feces and all feces in that period. A numerical score based on feces consistency was assigned as follows: normal feces = 1, semi-solid feces = 2, and watery feces = 3.

#### Castor oil-induced intestinal transit

Castor oil-induced intestinal transit was performed according to the previously described method [40]. The mice were assigned into four groups of five mice for each. Two groups were given the CTFAA (150 and 300 mg/kg) via the oral route. Another group was given loperamide hydrochloride (5 mg/kg) orally, and the control group was given 1% DMSO solution (10 ml/kg) orally. Thirty minutes later, all groups of mice were administered castor oil (0.2 ml/mouse, p.o). Thirty minutes after the administration of castor oil, the animals were given a standard charcoal meal (0.2 ml/mouse, made up of 5% charcoal suspension in distilled water) orally. Mice were killed with a lethal dose of chloroform before they were subjected to scarification. All the animals in each treatment group were killed after 30 min of the administration of the charcoal meal and the small intestine immediately isolated from pylorus to cecum. The peristaltic index (PI), which is the distance traveled by the charcoal meal relative to the total length of small intestine expressed in percentage, was determined for each mouse.

#### Castor oil-induced intestinal fluid accumulation

Castor oil-induced intestinal fluid accumulation was performed according to the previously described method [41]. Total 4-group with five mice in each were assigned in the study. Mice were pretreated with vehicle (10 ml/kg, p.o) or loperamide hydrochloride (5 mg/kg, p.o) and CTFAA (150 and 300 mg/kg, p.o). Thirty minutes after, the mice were given castor oil (0.2 ml/mouse) orally. Mice were killed with a lethal dose of chloroform before they were subjected to scarification. Mice were killed 30 min later of the treatment, and the small intestine was removed, after ligation at the pyloric end and ileocaecal junction, and weighed. The intestinal contents were then expelled into a graduated tube, and the volume determined. The small intestine was reweighed, and the difference between full and empty intestine was calculated.

### Ethical consideration

All experiments include animal works were taken place at the Department of Pharmacy, International Islamic University Chittagong, Bangladesh. Ethical approval was received for handling mice (for the present study) from the institutional ethical committee before starting the experiment. “Principles of the laboratory animal care” (NIH publication no. 85-23, revised 1985) and “national animal care laws” were strictly followed during handling of these animals for the study, for the human-related experiment was conducted in accordance with the ethical standards laid down in the 1964 Declaration of Helsinki. The study protocol was approved by the Department of Pharmacy, International Islamic University Chittagong, Bangladesh (Consent approval number: ECPD-IIUC-2018/04).

### Statistical analysis

The representation of the data was as mean ± standard error of the mean (SEM). One-way analysis of variance (ANOVA) followed by Tukey’s test and Dunnett’s test was used to describe the data for significant differences between the test and control groups. *P* values (<0.05 and <0.01) were considered as statistically significant. All statistical analysis was performed using SPSS v 16 and GraphPad Prism v 5.

## Results

### Toxicity study

**Figure 1:**
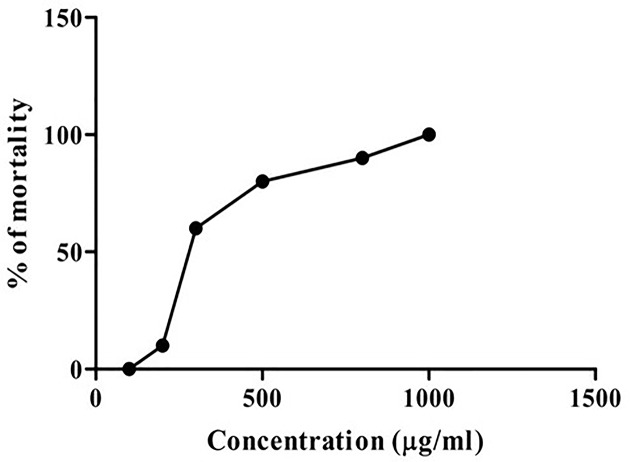
CTFAA activity in the brine shrimp lethality assay Various concentrations of CTFAA were assessed on the viability of brine shrimp nauplii after 24 h incubation (*n* = 10). Concentrations of CTFAA vs toxicity in brine shrimp nauplii were presented in the figure.

Cytotoxic effect of the CTFAA is summarized in Figure 1. A concentration–response relationship was found where the percentage of the mortality was increased gradually with increasing concentration. The LC_50_ for CTFAA and vincristine sulfate was found to be 424.16 and 0.74 μg/ml, respectively.

Any abnormal behavior or other signs of toxicity were not observed to the mice at several doses (1000, 2000, and 3000 mg/kg) in the acute toxicity study. There was also no mortality observed in the study period, suggesting that the LD_50_ of the CTFAA is higher than 3000 mg/kg. The doses were selected based on the results of the acute toxicity study. Considering the safety of the CTFAA, 1/10th of the maximum dose (3000 mg/kg) given in the toxicity study limit test was considered as a high dose (300 mg/kg) and half of the high dose assigned as a low dose (150 mg/kg).

### Antioxidant assay

#### DPPH radical scavenging activity

**Figure 2:**
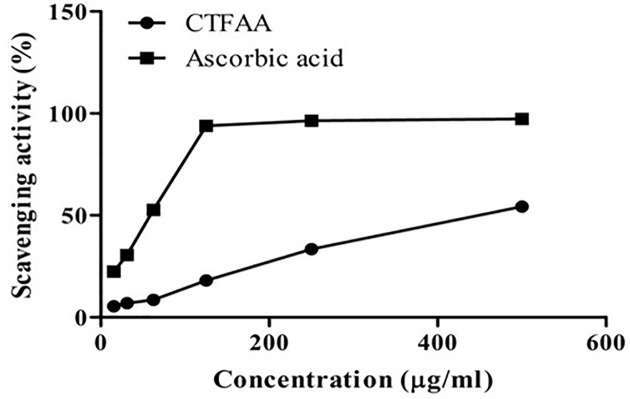
DPPH radical scavenging activity of CTFAA and ascorbic acid CTFAA dose-dependently scavenges the DPPH radical; ascorbic acid was used as the positive control.

The DPPH radical scavenging ability of CTFAA and ascorbic acid is shown in Figure 2. From Figure 2 we observed that a concentration–response relationship is found in the DPPH radical scavenging activity, the activity increased as the concentration increased for CTFAA and ascorbic acid, while the IC_50_ was found to be 446.04 and 55.31 μg/ml respectively.

#### Reducing power activity

**Figure 3:**
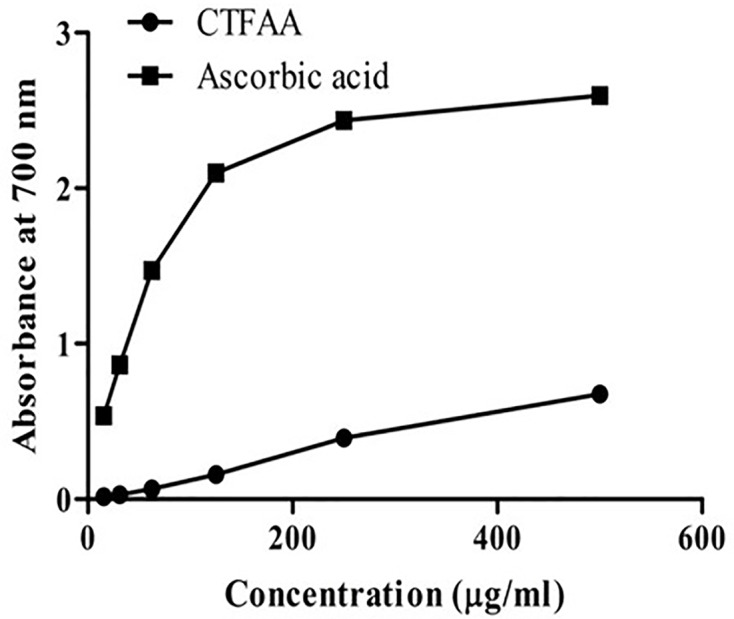
Reducing power of CTFAA Different concentration of CTFAA shows reducing power, and ascorbic acid was used as a positive control.

Figure 3 shows the reducing capacity of CTFAA and ascorbic acid at various concentrations. A concentration-dependent relationship was found, whereas reducing the power of CTFAA increased with increasing concentration as compared with ascorbic acid. The reductive capability is shown in the following order: ascorbic acid > CTFAA.

#### Total phenolics, flavonoids, and flavonols

**Figure 4:**
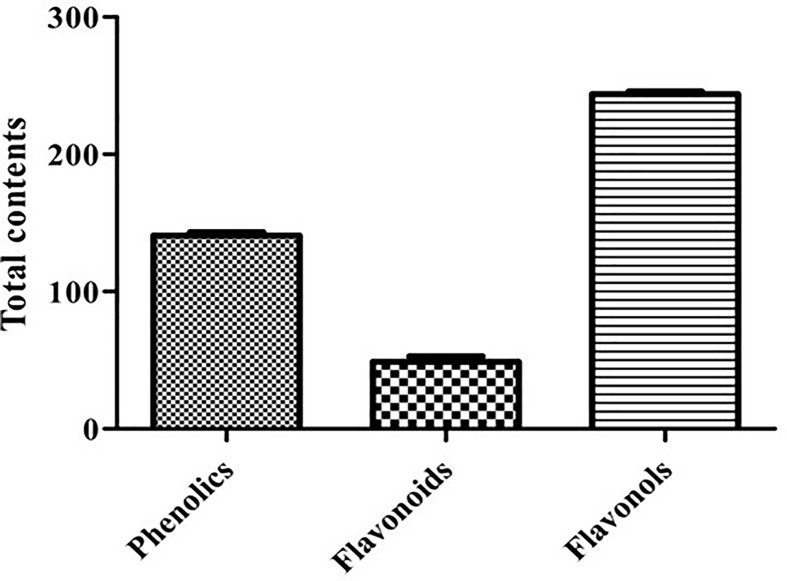
Total amount of phenolic, flavonoid, and flavonol contents of CTFAA Values are presented as the mean ± SEM (*n* = 3). Unit for the total phenolic is mg GAE/g, the flavonoid is mg QE/g, and flavonol is mg QE/g.

The result of total phenolic, flavonoids, and flavonols contents in CTFAA is shown in Figure 4. The total phenolic, flavonoid, and flavonol contents were found to be 140.77 ± 2.51 (mg GAE/g), 48.93 ± 3.89 (mg QE/g), and 243.94 ± 2.01 (mg QE/g), respectively.

### Thrombolytic activity

**Figure 5:**
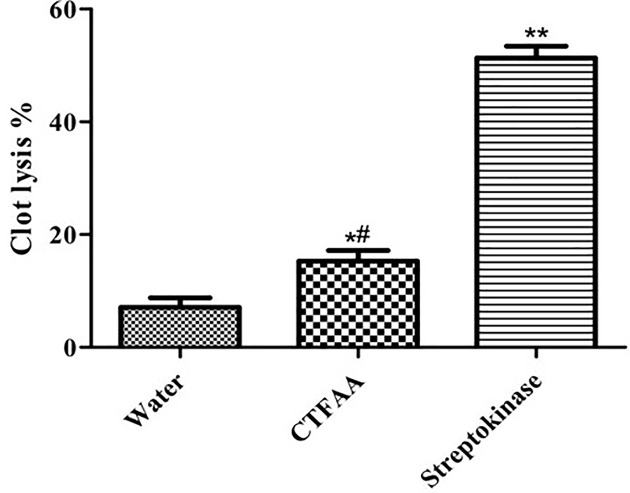
Clot lysis effect of CTFAA Values are presented as the mean ± SD (*n* = 10). *P<0.05 and **P<0.01 compared with the negative control group and ^#^*P*<0.05 compared with the positive control group (one-way ANOVA followed by Tukey’s test).

The thrombolytic activity of the CTFAA and appropriate controls have shown in Figure 5. Clots, when treated with distilled water (negative control), showed only negligible clot lysis (7.08%). Addition of SK, a positive control to the clots, showed 51.31% clot lysis. The mean difference in clot lysis percentage between positive and negative control was statistically very significant (*P*<0.01). After treatment of clots with CTFAA, 15.29% clot lysis was obtained, which was statistically significant (*P*<0.05) as compared with the positive and negative controls.

### Antidiarrheal activity

#### Castor oil-induced diarrhea

After administration of CTFAA (150 and 300 mg/kg), a dose-dependent significant (*P*<0.05) delay in the onset of diarrhea produced compared with control. Similarly, there was a reduction in the number of wet feces, total number of feces, weight of wet feces and total weight of all feces. Besides the data revealed that the percentage of diarrheal inhibitions was 16.27% and 41.81% at the doses of 150 and 300 mg/kg, respectively (Table 1). The reference standard drug loperamide also produced a significant (*P*<0.01) delay in initiation of diarrhea and 71.62% diarrheal inhibitions was obtained.

**Table 1:** Effect of CTFAA on castor oil-induced diarrhea in mice (*n* = 5)

Treatment	Dose (mg/kg)	The onset of diarrhea (min)	Number of wet feces	Total number of feces	Total weight of wet feces (g)	Total weight of all feces (g)	% Inhibition of defecation
Control	–	58.33 ± 2.69	9.16 ± 0.84	13.84 ± 0.32	0.44 ± 0.03	0.68 ± 0.03	–
Loperamide	5	219.34 ± 5.25**	2.60 ± 0.44**	2.18 ± 0.41**	0.14 ± 0.01**	0.15 ± 0.01**	71.62
CTFAA	150	75.67 ± 12.39*	7.67 ± 1.67*	11.67 ± 1.67*	0.39 ± 0.08*	0.51 ± 0.09*	16.27
CTFAA	300	104.67 ± 23.13*	5.33 ± 1.20*	10.67 ± 1.67*	0.33 ± 0.06*	0.48 ± 0.06*	41.81

Values are presented as the mean ± SEM. *P<0.05 and **P<0.01 compared with the control group (one-way ANOVA followed by Dunnett’s test).

#### Castor oil-induced intestinal transit

The CTFAA (150 and 300 mg/kg) caused significant (*P*<0.01) reduction in the distance traveled by the charcoal meal in the small intestine relative to the total length of the small intestine (PI), compared with the control. The percentage of inhibitions were 46.32% and 50.61% at doses of 150 and 300 mg/kg, respectively. A significant reduction (*P*<0.01) in the PI with 64.27% inhibition was also observed with the reference standard drug loperamide (Table 2).

**Table 2:** Effect of CTFAA on castor oil-induced intestinal transit in mice (*n* = 5)

Treatment	Dose (mg/kg)	Peristaltic index (%)	Inhibition (%)
Control	–	83.07	–
Loperamide	5	29.68**	64.27
CTFAA	150	44.59**	46.32
CTFAA	300	41.03**	50.61

Values are presented as the mean ± SEM. **P<0.01 compared with the control group (one-way ANOVA followed by Dunnett’s test).

#### Castor oil-induced intestinal fluid accumulation

In the fluid accumulation test, the CTFAA (150 and 300 mg/kg) significantly (*P*<0.05 and *P*<0.01) reduced both the volume and weight of intestinal contents, whereas 17.05%, 28.41%, 54.87%, and 57.52% inhibitions were obtained. Similarly, the reference standard drug loperamide also significantly (*P*<0.01) reduced both the volume and weight of intestinal contents, on the contrary, 45.45% and 66.37% inhibitions were achieved (Table 3).

**Table 3:** Effect of CTFAA on castor oil-induced intestinal fluid accumulation in mice (*n* = 5)

Treatment	Dose (mg/kg)	Volume of intestinal content (ml)	Inhibition (%)	Weight of intestinal content (g)	Inhibition (%)
Control	–	0.88 ± 0.08	–	1.13 ± 0.04	–
Loperamide	5	0.48 ± 0.03**	45.45	0.38 ± 0.04**	66.37
CTFAA	150	0.73 ± 0.18*	17.05	0.51 ± 0.10**	54.87
CTFAA	300	0.63 ± 0.09*	28.41	0.48 ± 0.05**	57.52

Values are presented as the mean ± SEM. *P<0.05 and **P<0.01 compared with the control group (one-way ANOVA followed by Dunnett’s test).

## Discussion

We used *in vitro* and *in vivo* experimental techniques to evaluate different pharmacological activities of CTFAA. Several methods can be used to investigate the antioxidant effect of plant extracts [42], and here we used a common *in vitro* test to assess the antioxidant potentiality of CTFAA. The antioxidant effect of CTFAA was evaluated based on DPPH free radical scavenging activity and reducing power capacity assay. As a stable free radical, DPPH has been widely accepted as a tool for estimating free radical scavenging activities of an antioxidant. To become a stable diamagnetic molecule, it can accept an electron or hydrogen radical [43]. In this present study, CTFAA showed substantial concentration-dependent free radical scavenging activity. Thus, the phytochemical constituents of CTFAA might be releasing hydrogen and that neutralizing free radicals to scavenge the potential oxidative stress.

The reducing power capacity shown by the fraction is also reflecting the antioxidant efficiency of CTFAA. This activity was assessed by using Fe^3+^ to Fe^2+^ reduction assay, where the yellow color of the test solution changes to different shades of green and blue based on the reducing capacity of sample [44]. We have found that the reducing power activity of CTFAA was increased in a concentration-dependent manner. It has been reported that phenolic compounds play an essential role in the reducing power capacity of extracts and fractions [45]. CTFAA may be consist of a wide range of phenolic, flavonoid, and flavanol contents. It should also be considered that lots phenolic and flavonoid derivatives are extracted in less polar solvent like CCl_4_, which need to be assessed by comparing chemical characterization of different fractions. Overall, current analysis has correlated an establishment between the DPPH and total phenolic content. These results are complying with the previous investigations [46], where it has been interpreted that the extract with higher phenolic content exhibits potential scavenging of DPPH free radical. Antioxidant activity of most medicinal plants is because of their redox properties due to the presence of different polyphenolic derivatives, which donates hydrogen, and acts as reducing agent and oxygen scavenger [26,47].

Moreover, Brine shrimp lethality bioassay is a typical assay to assess the toxicity of plant extract or compounds [48]. The cytotoxicity assay showed that CTFAA has less lethality with LC_50_ of 424.16 μg/ml, which indicates that this extract is safe to use in the animal model.

Thrombolytic drugs block the formation of thrombus by binding with plasmin and lyses fibrinogen and fibrin coagulation. The different report confirms that flavonoid derivatives of plant interferes in platelet aggregation and reduces the risk of thrombosis and cardiovascular disease [49]. The current study has evaluated the thrombolytic activity of CTFAA, where SK used as a positive control and sterile distilled water used as a negative control. We find that CTFAA significantly increased the lyses clot as compared with negative controls. This preliminary investigation result is directing towards advanced study with CTFAA in particular cardiovascular disease model [50] as blood clot is the most critical event in which the damaged expanses of the endothelial cell surface or blood vessel are clogged by the deposition of fibrin, platelets and tissue factor [51]. The presence of phytochemicals, including saponin, tannin, and alkaloids in the fractions are the probable responsible for demonstrating the thrombolytic activity [52,53].

We also employed castor oil-induced diarrheal models in mice to investigate the antidiarrheal efficacy of CTFAA. Our study demonstrated that CTFAA has a dose-dependent substantiality to reduce the frequency of defecation. Castor oil is a known agent that causes increased permeability of water and electrolyte into intestinal mucosa that results in a watery luminal content flow throughout the small and large intestine [54]. In castor oil-induced diarrhea, CTFAA showed a significantly decreased the frequency of purging (reduction of the number of wet feces) and decreased in the weight of wet feces with increasing the percent of inhibition of defecation. Determination of the percentage of inhibition has been based on the reduction of the frequency of wet feces and is a good marker of antidiarrheal activity. Besides, our results directly demonstrated an inhibition of castor oil-induced intestinal fluid accumulation (enteropooling) with a significant reduction of the weight and volume of intestinal contents. The mechanism might be involving the gastrointestinal motility, as well as water and electrolyte transport (decreasing Na^+^ and K^+^ absorption) across the intestinal mucosa [55]. Thus, the CTFAA possibly reduced diarrhea by increasing reabsorption of the electrolytes and water or by inhibiting induced intestinal accumulation of fluid just as the standard drug like loperamide. CTFAA also significantly suppressed the propulsion of charcoal marker at all tested doses in castor oil-induced intestinal transit or motility. This finding suggests that CTFAA can influence the peristaltic movement of the intestine, thereby indicating the importance to be included as a supplement to reduce motility in the diarrheal patient.

Phytochemicals such as flavonoids and polyphenols are accountable for the antidiarrheal activity [56]. Flavonoids can prevent intestinal motility, and water and electrolytes secretion [57]. Moreover, flavonoids can inhibit prostaglandin E2 induced intestinal secretion- and spasmogens-induced contraction and also inhibit the release of prostaglandins and autacoids [56]. Thereby, flavonoids as the inhibitors of prostaglandins biosynthesis are considered to delay castor oil-induced diarrhea [58]. Polyphenols also can show antidiarrheal property by interacting and preventing cytochrome P450 systems [59]. According to our data, CTFAA contains flavonoid and phenolic compounds that might be responsible for the substantial antidiarrheal activity.

## Conclusion

As per the data discussed, CTFAA displayed modest antioxidant effect. However, it showed significant antidiarrheal activity and potentially lyses blood coagulation at the safest dose. This preliminary data is warranting further study to understand the molecular mechanism of extract and its active chemical constituents.

## Abbreviations

CHT: Chittagong Hill Tracts
CTFAA: carbon tetrachloride (CCl4) fraction of leaves of *A. angustifolia*
DMSO: dimethyl sulfoxide
DPPH: 2,2-diphenyl-1-picrylhydrazyl
PI: peristaltic index
SK: streptokinase
tPA: tissue plasminogen activator
